# Bildgebung bei Kindern und Jugendlichen intraoperativ, bei ausgewählten Frakturen und im Verlauf nach konservativer und operativer Behandlung: Teil 2 der Ergebnisse einer bundesweiten Online-Umfrage der Sektion Kindertraumatologie der Deutschen Gesellschaft für Unfallchirurgie

**DOI:** 10.1007/s00113-021-01114-3

**Published:** 2021-12-16

**Authors:** Klaus Dresing, Francisco Fernandez, Peter Schmittenbecher, Kaya Dresing, Peter Strohm, Christopher Spering, Ralf Kraus, Sektion Kindertraumatologie der Deutschen Gesellschaft für Unfallchirurgie

**Affiliations:** 1 Klinik für Unfallchirurgie, Orthopädie und Plastische Chirurgie, Universitätsmedizin Göttingen, Robert-Koch-Str. 40, 37099 Göttingen, Deutschland; 2 Klinik für Unfallchirurgie und Orthopädie, Klinikum Bad Hersfeld, Bad Hersfeld, Deutschland; 3 Kindertraumatologie, Klinikum Stuttgart Olgahospital, Stuttgart, Deutschland; 4 Kinderchirurgische Klinik, Städtisches Klinikum Karlsruhe, Karlsruhe, Deutschland; 5 Darmstädter Kinderkliniken Prinzessin Margaret, Darmstadt, Deutschland; 6 Klinik für Orthopädie und Unfallchirurgie, Klinikum Bamberg, Bamberg, Deutschland; 7 Sektion Kindertraumatologie der Deutschen Gesellschaft für Unfallchirurgie, Berlin, Deutschland

**Keywords:** Intraoperatives Röntgen, Nachkontrollen, Kindesalter, Jugendalter, Strahlenschutz, Deutschlandweite Umfrage, Intraoperative x‑rays, Postoperative checks, Childhood, Adolescence, Radiation protection, Germany-wide survey

## Abstract

**Zusammenfassung:**

Die Indikation für Röntgenuntersuchungen bei pädiatrischen und jugendlichen Traumapatienten sollte ALARA (as low as reasonable achievable)  folgen. Aufgrund der Wirkung der Strahlung auf das wachsende sensible Gewebe dieser jungen Patienten sollte immer eine strenge Indikation zum Strahleneinsatz und bei Kontrollen nach Frakturversorgung gestellt werden.

**Methoden:**

Eine Online-Umfrage der Sektion Kindertraumatologie (SKT) der Deutschen Gesellschaft für Unfallchirurgie (DGU) vom 15.11.2019 bis 29.02.2020. Zielgruppen waren Unfall‑, Kinder- und Allgemeinchirurgen sowie Orthopäden.

**Ergebnisse:**

Teilnehmer: 788. Intraoperative Anwendungen: Kollimation 50 % immer, Postprocessing zur Vergrößerung 40 %, gepulstes Röntgen 47 % und 89 % keine kontinuierliche Fluoroskopie; 63 % Osteosynthese nie direkt auf dem Bildverstärker. Röntgenkontrollen nach Metallentfernung werden von 24 % nie verwendet. Nach operierter suprakondylärer Humerusfraktur werden Kontrollen bis zu 6 Mal durchgeführt. Nach distaler Radius-Grünholzfraktur verzichten 40 % auf weitere Röntgenkontrollen, nach konservativ versorgter Clavicularschaftfraktur 55 % auf weitere Kontrollen, andere röntgen mehrfach. Nach nicht dislozierter konservativ versorgter Tibiaschaftfraktur empfehlen 63 % eine radiologische Kontrolle nach einer Woche in zwei Ebenen, 24 % nach 2 Wochen, 37 % nach 4 Wochen und 32 % nach 6 Wochen.

**Diskussion:**

Die Analyse zeigt, dass es kein einheitliches radiologisches Management von Kindern und Jugendlichen mit Frakturen unter den Befragten gibt. Bei einigen Indikation für den Einsatz von Röntgenstrahlen scheint der Nutzen nicht evident zu sein. Das ALARA-Prinzip scheint nicht durchgängig beachtet zu werden.

**Schlussfolgerung:**

Vergleicht man die dokumentierten Ergebnisse der Umfrage mit den Konsensergebnissen der SKT zeigen sich Unterschiede.

## Abstract (en)

**Abstract:**

The indication for radiographic examinations in pediatric and adolescent trauma patients should follow ALARA (as low as reasonably achievable). Because of the effect of radiation on the growing sensitive tissues of these young patients, a strict indication should always be given for radiation use and during controls after fracture repair.

**Methods:**

An online survey by the Pediatric Traumatology Section (SKT) of the German Trauma Society (DGU) from Nov. 15, 2019, to Feb. 29, 2020, targeting trauma, pediatric, and general surgeons and orthopedic surgeons.

**Results:**

Participants: 788. Intraoperative applications: Collimation 50% always, postprocessing for magnification 40%, pulsed x-ray 47%, and 89% no continuous fluoroscopy; 63% osteosynthesis never directly on image intensifier. Radiographic controls after implant removal never used by 24%. After operated supracondylar humerus fracture, controls are performed up to 6 times. After distal radius greenstick fracture, 40% refrain from further X-ray controls, after conservatively treated clavicular shaft fracture, 55% refrain from further controls, others X-ray several times. After nondisplaced conservatively treated tibial shaft fracture, 63% recommend radiographic control after 1 week in two planes, 24% after 2 weeks, 37% after 4 weeks, and 32% after 6 weeks.

**Discussion:**

The analysis shows that there is no uniform radiological management of children and adolescents with fractures among the respondents. For some indications for the use of radiography, the benefit does not seem evident. The ALARA principle does not seem to be consistently followed.

**Conclusion:**

Comparing the documented results of the survey with the consensus results of the SKT, differences are apparent.

## Hintergrund

Auch bei Verletzten mit Frakturen im Kindes- und Jugendalter ist die Röntgendiagnostik seit Jahrzehnten Standardverfahren. Intraoperativ ist auch in der Kindertraumatologie das Röntgen etabliert. Den Vorteilen dieser Diagnostik stehen die potenziellen Nachteile durch Strahlenschäden gegenüber.

Die Sektion Kindertraumatologie (SKT) der DGU wollte mit dieser Umfrage den Status quo in der intraoperativen und postoperativen Bildgebung und in der Nachkontrolle von ausgesuchten knöchernen Verletzungen und im Kindes- und Jugendalter erfragen. Wie sehen die in der Kindertraumatologie tätigen Ärzte und Ärztinnen die Notwendigkeit zur bildgebenden Diagnostik im OP und in der Nachkontrolle? Wie werden Maßnahmen zur Strahlenreduktion im OP eingeschätzt und umgesetzt?

## Methodik

Mit dem Programm SurveyMonkey (momentive ai, Europe UC Dublin, Irland) [63] wurde die Umfrage über den Einsatz der Röntgenstrahlen bei Kindern und Jugendlichen über das Portal der Deutschen Gesellschaft für Orthopädie und Unfallchirurgie (DGOU) durchgeführt. Zusätzlich wurden über den E‑Mail-Verteiler der Fachgesellschaft Kolleginnen und Kollegen aus Unfallchirurgie, Unfallchirurgie und Orthopädie, Orthopädie, Kinderchirurgie und Kinderorthopädie auf die Umfrage über den Einsatz der Röntgenstrahlen bei Kindern und Jugendlichen hingewiesen. Als Instrument wurde SurveyMonkey benutzt. Die Umfrage war freiwillig und erfolgte anonym.

Neben epidemiologischen Fragen wie Tätigkeitsprofilen, Weiterbildungen, wurden Fragen zur Häufigkeit der Versorgung und primären Diagnostik von Frakturen und Verletzungen in der Altersgruppe gestellt.

Die Auswertfunktion des Umfrageprogramms lieferte anonymisierte Daten im Excelformat. Die weitere Datenanalyse erfolgte mit dem Datenbankprogramm Filemaker [18] und mit JASP [65]. Ermittelt wurden Daten der zentralen Tendenz und prozentuale Verteilungen.

## Ergebnisse

### Intraoperative Einblendung beim Röntgen

Der Einsatz der Kollimation beim intraoperativen Röntgen wird unterschiedlich praktiziert^1^. Der Anteil der Befragten, die die Einblendung/Kollimation immer einsetzen, liegt knapp über 50 %. 6,7 % aller Befragten setzen die Blende zur Strahlenreduktion nie, fast 12 % selten ein; Tab. 1. Auffallend ist, dass die Praxis der Assistenten hier abweicht. Bei ihnen zeigt sich ein Anteil von 14,71 %, die die Blende nie benutzen. Die Kollegen in der Niederlassung verwenden die Einblendung in 66 %, 9,6 % nutzen sie nie.

**Table 1:** 

	Gesamt	CH	KiCH	O + U	SUCH	CA	OA	ASS	NL
Anzahl	759	289	143	520	277	171	295	127	166
Nie (in %)	6,72	3,81	6,29	6,92	3,97	3,51	3,73	14,17	9,64
Selten (in %)	11,73	7,96	9,79	12,31	12,27	8,19	12,88	16,54	9,64
Häufig (in %)	30,83	30,45	30,07	31,54	33,57	31,58	40,68	27,56	15,06
Immer (in %)	50,72	57,79	53,85	49,23	50,18	56,73	42,71	41,73	65,66

*CH* Chirurgie, *KiCH* Kinderchirurgie, *O* *+* *U* Orthopädie und Unfallchirurgie, *SUCH* spezielle Unfallchirurgie, *CA* Chefarzt, *OA* Oberärzte, *ASS* Assistenten, *NL* Niedergelassene

### Intraoperative Vergrößerung der Röntgenbilder

Um Röntgenbilder oder Ausschnitte intraoperativ zu vergrößern, wenden 40 % der Befragten das „postprocessing“ an, also ohne neue Röntgenaufnahmen zu generieren. 33 % verändern den Abstand zwischen Röhre und Bildverstärker, röntgen also erneut; Tab. 2. 26 % nutzen die intraoperative Möglichkeit der Vergrößerung nicht. Assistenten mit 54 % und Kinderchirurgen mit 51 % nutzen die Abstandsveränderungen zur intraoperativen Vergrößerung der Bildwandlerbilder.

**Table 2:** 

	Gesamt	CH	KiCH	O + U	SUCH	CA	OA	ASS	NL
Anzahl	733	289	143	520	277	171	295	127	166
Postprocessing (in %)	40,38	36,49	31,43	33,67	38,91	41,21	36,08	25,60	26,32
Abstandsveränderung (in %)	33,42	32,98	50,71	39,24	35,27	35,76	42,27	53,60	30,92
Keine (in %)	26,19	30,53	17,86	27,09	25,82	23,03	21,65	20,80	42,76

*CH* Chirurgie, *KiCH* Kinderchirurgie, *O* *+* *U* Orthopädie und Unfallchirurgie, *SUCH* spezielle Unfallchirurgie, *CA* Chefärzte, *OA* Oberärzte, *ASS* Assistenten, *NL* Niedergelassene

### Einsatz der gepulsten Röntgentechnik

Zur Dosisreduktion haben heutige Bildverstärker eine Einstellung zur digital gepulsten Durchleuchtung. 759 Befragte beantworteten diese Frage. In der Gesamtgruppe setzen 17,13 % diese Technik nie, 14,89 % selten, 23,32 % häufig und 44,68 % immer ein; Tab. 3. Bei den Kollegen in der Niederlassung wird das gepulste Röntgenverfahren in 42 % nie verwendet. 7 % in der Kinderchirurgie nutzen diese Technik nicht, 56 % verwenden sie in der Kinderchirurgie regelmäßig.

**Table 3:** 

	Gesamt	CH	KiCH	O + U	SUCH	CA	OA	ASS	NL
Anzahl	759	289	143	520	277	171	295	127	166
Nie (in %)	17,13	16,96	6,99	18,65	12,64	6,43	8,14	21,26	40,96
Selten (in %)	14,89	9,00	17,48	14,04	10,11	7,60	17,97	25,20	9,04
Häufig (in %)	23,32	25,61	19,58	25,00	29,60	31,58	23,39	22,05	15,66
Immer (in %)	44,66	48,44	55,94	42,31	47,65	54,39	50,51	31,50	34,34

*CH* Chirurgie, *KiCH* Kinderchirurgie, *O* *+* *U* Orthopädie und Unfallchirurgie, *SUCH* spezielle Unfallchirurgie, *CA* Chefärzte, *OA* Oberärzte, *ASS* Assistenten, *NL* Niedergelassene

### Fluoroskopie

Bei der Frage, ob intraoperativ die kontinuierliche Röntgendiagnostik (Fluoroskopie) eingesetzt wird, z. B. um Gelenke besser zu beurteilen können, antworteten knapp 90 % der Befragten, die Fluoroskopie nie oder selten einzusetzen; Tab. 4. Die niedergelassenen Ärzte geben an, in über 90 % die Fluoroskopie nie oder selten zu verwenden.

**Table 4:** 

	Gesamt	CH	KiCH	O + U	SUCH	CA	OA	ASS	NL
Anzahl	759	289	143	520	277	171	295	127	166
Nie (in %)	43,87	47,06	34,27	45,19	42,60	39,77	36,61	42,52	62,05
Selten (in %)	45,32	40,14	58,74	43,85	43,68	45,61	51,53	49,61	30,72
Häufig (in %)	8,30	10,38	4,20	8,46	10,83	9,94	9,83	7,09	4,82
Immer (in %)	2,50	2,42	2,80	2,50	2,89	4,68	2,03	0,79	2,41

*CH* Chirurgie, *KiCH* Kinderchirurgie, *O* *+* *U* Orthopädie und Unfallchirurgie, *SUCH* spezielle Unfallchirurgie, *CA* Chefärzte, *OA* Oberärzte, *ASS* Assistenten, *NL* Niedergelassene

### Osteosynthesen direkt auf dem Bildverstärker

Es ist in einigen Praxen und Krankenhäusern üblich, Osteosynthesen bei Kindern und Jugendlichen direkt auf dem steril abgedeckten Bildverstärker („auf dem Topf“) durchzuführen.

Im Krankenhaus werden in 66 % nie, im Niedergelassenenbereich zu 51 % nie Operationen direkt auf dem Bildverstärker durchgeführt.

63 % aller Befragten operieren nie, 22 % selten auf dem Bildverstärker. 5 % praktizieren diese Technik regelhaft; Tab. 5. Bei den Niedergelassenen werden in 8 % Osteosynthesen regelhaft „auf dem Topf“ ausgeführt.

**Table 5:** 

	Gesamt	CH	KiCH	O + U	SUCH	CA	OA	ASS	NL
Anzahl	725	279	139	497	273	164	290	124	147
Nie (in %)	63,03	62,72	61,87	62,58	64,10	64,02	66,55	66,13	52,38
Selten (in %)	22,21	25,45	16,55	23,54	26,01	21,34	21,72	15,32	29,93
Häufig (in %)	9,52	8,60	15,83	8,45	7,69	8,54	9,31	11,29	9,52
Regelhaft (in %)	5,24	3,23	5,76	5,43	2,20%	6,10	2,41	7,26	8,16

*CH* Chirurgie, *KiCH* Kinderchirurgie, *O* *+* *U* Orthopädie und Unfallchirurgie, *SUCH* spezielle Unfallchirurgie, *CA* Chefärzte, *OA* Oberärzte, *ASS* Assistenten, *NL* Niedergelassene

## Bildgebung bei einer Auswahl häufiger Frakturen und Maßnahmen

### Röntgenbildgebung nach Metallentfernung

Abgefragt wurden verschiedene Röntgenoptionen nach einer Metallentfernung. Allgemein wird von allen Befragten in 12 % eine Röntgendokumentation bei allen Patienten gewünscht. Hier zeigen sich Assistenten mit 16 % und Niedergelassene mit 14 % im oberen Bereich; Tab. 6. Bei Patienten im Rahmen des BGlichen Heilverfahrens wurden in 4,3 % Röntgenuntersuchungen gefordert. Hier ist bei den Kollegen in der Niederlassung dieser Anteil bei 10 %. Wenn postoperativ nach Metallentfernung geröntgt wird, begnügen sich 12,5 % mit einer Ebene, 5 % fordern eine zweite Ebene. Für 23 % ist die intraoperative Röntgenbildgebung mit dem Bildverstärker immer ausreichend, für 31 % häufig, für 6 % gelegentlich ausreichend. Während 25 % kein postoperatives Röntgenbild für angebracht halten, liegt der Wert bei den Kinderchirurgen bei 67 %.

**Table 6:** 

	Gesamt	CH	KiCH	O + U	SUCH	CA	OA	ASS	NL
Anzahl	727	281	137	498	273	163	289	125	150
Konv. Rö. (BG-Patienten) (in %)	4,26	5,69	1,46	4,42	4,76	3,68	1,73	4,00	10,00
Konv. Rö. alle Patienten (in %)	11,97	10,68	5,11	12,65	13,19	9,20	10,73	16,00	14,00
Vor Entl. konv. Rö. 1 Eb. (in %)	12,52	13,88	5,11	15,06	16,12	17,18	11,76	12,80	8,67
Vor Entl. konv. Rö. 2 Eb. (in %)	5,09	7,12	3,65	6,02	6,59	6,75	4,84	4,80	4,00
I‑op. BV-Bild immer ausreichend (in %)	23,25	25,98	8,76	26,31	25,64	24,54	25,26	21,60	19,33
I‑op. BV-Bild häufig ausreichend (in %)	31,50	34,52	13,87	37,55	37,00	30,67	32,18	28,80	33,33
I‑op. BV-Bild gelegentl. ausreichend (in %)	6,05	6,05	5,84	6,43	5,13	5,52	6,23	4,80	7,33
Kein pop. Röntgen (in %)	24,48	19,57	67,15	13,45	15,38	26,99	24,22	23,20	23,33

*CH* Chirurgie, *KiCH* Kinderchirurgie, *O* *+* *U* Orthopädie und Unfallchirurgie, *SUCH* spezielle Unfallchirurgie, *CA* Chefärzte, *OA* Oberärzte, *ASS* Assistenten, *NL* Niedergelassene, *i‑op.* intraoperativ, *pop.* postoperativ, *BG* berufsgenossenschaftliche (Patienten), *BV* Bildverstärker, *Rö*. Röntgen, *Entl*. Entlassung, *Eb*. Ebenen

### Bildgebung nach Bohrdrahtosteosynthese einer suprakondylären Humerusfraktur

Die Fragestellung richtete sich hier auf die Röntgenbildgebung im Verlauf nach der Osteosynthese bis zur Metallentfernung. Direkt postoperativ röntgen 32 %. Kinderchirurgen sehen eine Indikation hälftig weniger mit 15 %; Tab. 7. 46 % aller Niedergelassenen wollen eine Röntgenkontrolle direkt postoperativ. 30 % sehen eine Röntgenaufnahme vor der Entlassung indiziert. Die Gruppe der Assistenten sieht mit 10 % mehr, also 41 % diese Röntgendokumentation als notwendig an. Eine Woche postoperativ wünschen 18 % insgesamt eine Röntgenkontrolle. Nach 2 Wochen röntgen 10 %, nach 4 Wochen 23 %, nach 6 Wochen 14,5 % der Befragten. Vor der Metallentfernung fordern 30 % eine Kontrolle. 33 % aller sehen eine Indikation bei dieser Versorgung nur zur postoperativen Röntgenuntersuchung und vor der Metallentfernung.

**Table 7:** 

	Gesamt	CH	KiCH	O + U	SUCH	CA	OA	ASS	NL
Anzahl	702	275	136	479	273	160	285	120	137
Direkt pop. (in %)	32,34	39,27	15,44	37,58	36,26	31,87	30,53	21,67	45,99
Pop vor Entl. (in %)	29,91	25,09	26,47	31,32	28,21	30,63	28,42	40,83	22,63
N. 1 Wo. (in %)	18,09	20,73	11,76	19,62	21,25	17,50	20,70	10,83	19,71
N. 2 Wo. (in %)	10,11	10,55	2,21	11,69	13,19	13,13	8,07	8,33	12,41
N. 4 Wo. (in %)	22,65	25,45	27,94	22,34	23,81	25,00	24,21	14,17	24,09
N. 6 Wo. (in %)	14,53	14,18	2,94	17,54	16,12	15,63	14,39	12,50	15,33
Vor ME (in %)	31,34	30,91	25,00	33,61	37,36	30,00	34,04	31,67	27,01
Pop + vor ME (in %)	32,91	33,82	37,50	31,52	30,40	31,87	30,18	40,83	32,85

*CH* Chirurgie, *KiCH* Kinderchirurgie, *O* *+* *U* Orthopädie und Unfallchirurgie, *SUCH* spezielle Unfallchirurgie, *CA* Chefärzte, *OA* Oberärzte, *ASS* Assistenten, *NL* Niedergelassene, *pop* postoperativ, *ME* Metallentfernung, *Wo*. Woche(n)

### Bildgebung nach Grünholzfraktur des distalen Radius eines 6- bis 10-jährigen Kindes

702 der Befragten beantworteten diese Frage. Bei Betrachtung der Gesamtbefragung zeigt sich, dass 4 % nach einer Woche eine Ebene röntgen. 37 % wollen zu diesem Zeitpunkt 2 Röntgenebenen. Nach 2 Woche fordern 1,6 % eine Ebene, 11 % 2 Ebenen; Tab. 8. Nach 4 Wochen wollen 1,4 % eine Ebene, 28 % zwei Ebenen röntgen. Insgesamt verzichten 40 % auf jegliche Röntgenkontrolle. Niedergelassene führen in dieser Kategorie (keine Röntgen) mit 49 % vor den Unfallchirurgen mit 45 % durch.

**Table 8:** 

	Gesamt	CH	KiCH	O + U	SUCH	CA	OA	ASS	NL
Anzahl	702	275	136	479	272	159	285	120	138
1 Wo. in 1 Eb. (in %)	3,99	2,55	3,68	4,18	4,04	5,03	4,56	3,33	2,17
1 Wo. in 2 Eb. (in %)	37,46	30,91	47,06	35,49	33,46	33,33	40,35	46,67	28,26
2 Wo. in 1 Eb. (in %)	1,57	1,45	0,74	2,09	1,84	1,26	1,05	1,67	2,90
2 Wo. in 2 Eb. (in %)	10,68	8,00	5,15	12,32	9,93	9,43	11,58	9,17	11,59
4 Wo. in 1 Eb. (in %)	1,42	0,00	0,74	1,88	1,84	1,26	1,40	0,83	2,17
4 Wo. in 2 Eb. (in %)	28,06	27,27	30,15	28,81	24,63	31,45	24,56	30,83	28,99
Keine (in %)	40,03	46,91	38,97	39,04	43,01	39,62	39,30	32,50	48,55

*CH* Chirurgie, *KiCH* Kinderchirurgie, *O* *+* *U* Orthopädie und Unfallchirurgie, *SUCH* spezielle Unfallchirurgie, *CA* Chefärzte, *OA* Oberärzte, *ASS* Assistenten, *NL* Niedergelassene, *pop* postoperativ, *ME* Metallentfernung, *Wo*. Woche(n), *Eb*. Ebenen

### Röntgenuntersuchungen bei der konservativen Behandlung einer diaphysären Claviculafraktur eines 6- bis 10-jährigen Kindes

10,4 % der Befragten gaben an, nach einer Woche eine Ebene, 14,4 % zwei Ebenen zu röntgen. 5,8 % sahen nach 2 Wochen eine Röntgenebene, 7 % zwei Ebenen als indiziert an. Nach 4 Wochen fordern 9,4 % eine Ebene, 12,5 % zwei Ebenen der Clavicula; Tab. 9. Insgesamt wollen 54,8 % aller Befragten keine Röntgenkontrolle bei dieser Fraktur durchführen. 86,8 % aller Kinderchirurgen verzichten auf jegliche Röntgenkontrollen bei konservativer Behandlung einer diaphysären Claviculafraktur.

**Table 9:** 

	Gesamt	CH	KiCH	O + U	SUCH	CA	OA	ASS	NL
Anzahl	702	275	136	480	273	160	285	119	138
1 Wo. 1Eb. (in %)	10,40	10,07	5,80	12,16	9,49	11,25	6,32	15,13	13,77
1 Wo. 2Eb. (in %)	14,39	12,59	1,45	18,24	15,69	11,25	16,84	19,33	8,70
2 Wo. 1Eb. (in %)	5,84	6,12	1,45	5,03	7,66	5,63	4,91	4,20	9,42
2 Wo. 2Eb. (in %)	6,70	4,68	1,45	5,66	5,84	5,63	5,96	8,40	7,97
4 Wo. 1Eb. (in %)	9,40	7,91	4,35	7,76	6,20	11,25	4,91	2,52	22,46
4 Wo. 2Eb. (in %)	12,54	4,68	0,00	5,24	3,28	10,00	10,18	22,69	11,59
ø (in %)	54,84	53,96	85,51	45,91	51,82	57,50	60,00	47,06	47,83

*CH* Chirurgie, *KiCH* Kinderchirurgie, *O* *+* *U* Orthopädie und Unfallchirurgie, *SUCH* spezielle Unfallchirurgie, *CA* Chefärzte, *OA* Oberärzte, *ASS* Assistenten, *NL* Niedergelassene, *Wo*. Woche(n), *Eb*. Ebenen

Aus der detaillierten Analyse, wie viele Befragte – kategorisiert nach Facharztqualifikation – zu den angegebenen Zeitpunkten einmalig röntgen oder wie viele noch zusätzliche Untersuchungen ansetzen, ist zu erkennen, dass einige Gruppen auch Indikation zu weiteren Röntgenuntersuchungen bei der konservativ behandelten Claviculafraktur als notwendig ansehen; Tab. 10.

**Table 10:** 

	CH		KiCH		O + U		SUCH	
Anzahl	275	–	136	–	480	–	273	–
Zeitpunkt	–	+RöU	–	+RöU	–	+RöU	–	+RöU
1 Wo. 1 Eb.	10,07 %	0,72 %: 2 Wo. 1 Eb.	5,80 %	–	12,16 %	0,42 %: 2 Wo. 1 Eb.	9,49 %	0,73 %: 2 Wo. 1 Eb.
		0,72 %: 2 Wo. 1 Eb. + 4 Wo. 1 Eb.	–	–		0,42 %: 2 Wo. 1 Eb. + 4 Wo. 2 Eb.	–	0,36 %: 2 Wo. 1 Eb. + 4 Wo. 1 Eb.
		1,08 %: 4 Wo. 1 Eb.	–	–		1,68 %: 2 Wo. 2 Eb. + 4 Wo. 2 Eb.	–	–
				–		1,47 %: 4 Wo. 1 Eb.	–	–
				–		0,63 %: 4 Wo. 2 Eb.	–	–
1 Wo. 2 Eb.	12,59 %	0,72 %: 2 Wo. 2 Eb.	1,45 %	–	18,24 %	1,05 %: 2 Wo. 2 Eb.	15,69 %	1,46 %: 2 Wo. 2 Eb.
		1,80 %: 4 Wo. 2 Eb.	–	–		6,92 %: 4 Wo. 2 Eb.	–	0,73 %: 2 Wo. 2 Eb. + 4 Wo. 2 Eb.
		0,36 %: 2 Wo. 2 Eb. + 4 Wo. 2 Eb.	–	–		1,68 %: 2 Wo. 2 Eb. + 4 Wo. 2 Eb.	–	–
2 Wo. 1 Eb.	6,12 %	0,72 %: 4 Wo. 1 Eb.	1,45 %	–	5,03 %	0,84 %: 4 Wo. 1 Eb.	7,66 %	0,73 %: 4 Wo. 1 Eb.
				–		0,42 %: 4 Wo. 2 Eb.	–	–
2 Wo. 2 Eb.	4,68 %	0,72 %: 4 Wo. 2 Eb.	1,45 %	–	5,66 %	0,42 %: 4 Wo. 2 Eb.	5,84 %	1,82 %: 4 Wo. 2 Eb.
4 Wo. 1 Eb.	7,91 %	–	4,35 %	–	7,76 %	–	6,20 %	–
4 Wo. 2 Eb.	4,68 %	–	0,00 %	–	5,24 %	–	3,28 %	–
ø	53,96 %	–	85,51 %	–	45,91 %	–	51,82 %	–

*CH* Chirurgie, *KiCH* Kinderchirurgie, *O* *+* *U* Orthopädie und Unfallchirurgie, *SUCH* spezielle Unfallchirurgie, *+RöU* weitere Röntgenuntersuchungen, *Wo* Wochen, *Eb.* Ebenen

### Röntgenverlaufskontrollen nach undislozierter, isolierter konservativ behandelter Tibiaschaftfraktur

Nach undislozierter, isolierter Tibiaschaftspiralfraktur und konservativer Behandlung eines 6‑ bis 10-jährigen Kindes fordern die Kollegen überwiegend Röntgenkontrollen in 2 Ebenen, nach einer Woche 63 %, nach 2 Wochen 24 %, nach 4 Wochen 37 % und nach 6 Wochen 32 %. Auf die Röntgenkontrolle verzichten 5,4 % aller Befragten komplett; Tab. 11.

**Table 11:** 

	Gesamt	CH	KiCH	O + U	SUCH	CA	OA	ASS	NL
Anzahl	700	275	136	478	273	160	284	118	138
1 Wo. 1 Eb. (in %)	3,14	4,36	2,21	3,35	3,66	4,38	3,52	0,00	3,62
1 Wo. 2 Eb. (in %)	63,00	56,00	69,12	62,13	59,71	51,25	70,42	72,88	52,90
2 Wo. 1 Eb. (in %)	1,57	1,09	0,74	1,88	2,20	2,50	1,06	85,00	2,17
2 Wo. 2 Eb. (in %)	24,14	25,09	9,56	25,94	27,84	24,38	24,30	27,97	20,29
4 Wo. 1 Eb. (in %)	3,14	5,45	1,47	3,35	5,49	5,00	21,10	0,85	5,07
4 Wo. 2 Eb. (in %)	36,71	33,82	53,68	33,47	31,50	35,63	38,73	26,27	42,75
6 Wo. 1 Eb. (in %)	1,29	0,73	0,00	1,46	1,47	0,00	1,06	0,85	3,62
6 Wo. 2 Eb. (in %)	32,14	34,18	13,97	38,82	35,90	30,63	34,15	33,90	28,26
Ø (in %)	5,43	6,91	5,15	5,23	5,49	6,88	4,23	6,78	5,07

*CH* Chirurgie, *KiCH* Kinderchirurgie, *O* *+* *U* Orthopädie und Unfallchirurgie, *SUCH* spezielle Unfallchirurgie, *CA* Chefärzte, *OA* Oberärzte, *ASS* Assistenten, *NL* Niedergelassene, *Wo*. Woche(n), *Eb*. Ebene(n)

## Diskussion

Intraoperative Röntgenbildgebung ist auch bei der Versorgung von Frakturen im Kindes- und Jugendalter genauso Standard wie bei der Versorgung von Erwachsenen [30, 33, 44, 55]. Die Vorteile und Erfolge dieser Bildgebung sind anerkannt, es bedarf aber auch heute immer einer rechtfertigenden Indikation, auch zur Röntgendiagnostik. Der kindliche Organismus ist im Vergleich zum Erwachsenen strahlensensibler und damit anfälliger, durch ionisierende Strahlen Malignome zu entwickeln, auch weil die jungen Patienten eine längere Lebenserwartung und damit Zeitspanne haben [7, 30, 41, 46].

Bei Kindern soll besonders auf die jeweilige individuelle Abwägung zwischen Nutzen und Risiko beim Einsatz dieser Technik Wert gelegt werden. Dem ALARA-Prinzip sollte in dieser Altersgruppe im OP und in der Nachsorge konsequent gefolgt werden [19, 28, 30, 46, 71]. Auch bei der Indikation zur intraoperativen Röntgenuntersuchung und in der postoperativen oder posttraumatischen Verlaufskontrolle sollten immer individuell Nutzen und Risiko abgewogen werden [71].

Medikolegaler und Druck der Erziehungsberechtigten auf die behandelnden Kindertraumatologen, Röntgenaufnahmen zu veranlassen, sollten in den Hintergrund gestellt werden. Aber es muss auch das Wissen über Strahlenvermeidung vertieft werden [5].

Die intraoperative Strahlenhygiene sollte insbesondere bei Kindern und Jugendlichen oberste Priorität haben. Auch bei jedem Einsatz von ionisierender Strahlung während der Operationen muss jede Möglichkeit der Strahlenreduktion ergriffen werden. Dies beginnt schon mit der Auslösung der Strahlung am Bildverstärker. Es zeigt sich eine signifikante Reduktion der Strahlung, wenn der Operateur die Strahlung mit dem Fußschalter selbst auslöst, im Vergleich zur Auslösung der Strahlung durch andere Akteure [57]. Die Positionierung des C‑Bogens kann mithilfe der integrierten Laservorrichtung ohne Röntgen relativ präzise orientiert an anatomischen Landmarken erfolgen. Mithilfe digitaler Positionierungssystem können ebenfalls die Hauteintrittsdosis und die Streustrahlung nach einer Studie um 55 % bzw. 70 % reduziert werden [15]. Panchbhavi et al. bestätigten eine signifikante Genauigkeit der Positionierung und Abnahme der Röntgenbildanzahl beim Lasereinsatz [45].

### Kollimation

Durch Einblendung mittels Längs- oder Irisblende, die sog. Kollimation, lassen sich das Röntgenfeld und damit die Strahlenbelastung reduzieren, da nur der interessierende anatomische Bereich und damit die optimale Feldgröße eingestellt werden. Es kommt zu einer Verringerung des bestrahlten Körpervolumens und damit zu weniger Streustrahlung [56]. Voraussetzung ist, dass vorher mit dem Laser die möglichst exakte Position eingestellt wurde. Eine Suche der „region of interest“ unter Röntgen mit einem kleinen eingeblendeten Feld ohne Laser-Positionierung führt zu höherer Strahlung.

Unter normalen Bedingungen sind deterministische Schäden hier weniger zu erwarten [22]. Das Risiko von stochastischen Langzeiteffekten bleibt jedoch ein Thema, auch bei den relativ niedrigen eingesetzten Dosen [22]. In den Strahlenschutzregularien in Großbritannien ist festgehalten, dass es besonders bei Kindern wichtig ist, dass das Feld auf den wesentlichen Bereich beschränkt ist. Dieses Feld sollte immer kleiner als die Detektorgröße sollte, um die Kollimation zu einzuhalten [6]. Aus Sicht des Strahlenschutzes ist eine zu weite Kollimation besonders ungünstig, da sie zu einer unnötigen Exposition des Körpers außerhalb der interessierenden Region führt. Durch Kollimation lässt sich bei allen radiologischen Verfahren die Strahlenbelastung reduzieren [3, 4, 69, 72]. Mit Kollimation kann die Strahlung bei kontinuierlicher Fluoroskopie bis zu 75,6 % reduziert werden [72].

Über 18 % der Befragten nutzen diese Einstellung nie oder selten. 31 % verwenden die Apertur häufig, 51 % immer. Die SKT empfiehlt die großzügige Verwendung der intraoperativen Einblendung [16].

### Fluoroskopie

*Fluoroskopie* ist das kontinuierliche Röntgen, die Durchleuchtung, mit dem C‑Bogen, teilweise auch als Videofluoroskopie bezeichnet. Mit der Fluoroskopie können dynamische Untersuchungen, z. B. zur Aufklappbarkeit von Gelenken eingesetzt, erfolgen. Die Informationen dieser Untersuchung können fast immer auch aus Einzelaufnahmen erhalten werden. Bereits 1998 sahen Jones und Stoddart die Fluoroskopie als komplett vermeidbar an [29]. Zumindest sollte diese Technik sehr zurückhaltend eingesetzt werden [54]. Die Strahlenbelastung bei der kontinuierlichen Röntgendiagnostik, auch als „real-time moving fluoroscopy“ bezeichnet, wird unterschätzt [60].

Beim Wechsel von Fluoroskopie (kontinuierlichem Röntgen) zu gepulster Röntgentechnik konnte die Dosis bei perkutanen Eingriffen an der Wirbelsäule um 97,3 % reduziert werden [51]. Beim Vergleich von kontinuierlicher Fluoroskopie mit 8‑mal/s gepulster Technik zeigte sich eine Reduzierung der Strahlung auf 37,5 % [72]. Nach dem Konsens der SKT sollte die intraoperative Fluoroskopie auf ein Minimum reduziert werden [16].

### Vergrößerung

Die *Vergrößerung* wird häufig eingesetzt, um die kleinen Knochen bildfüllend darzustellen. Dies findet durchaus häufiger bei Kindern wegen der kleineren Knochen Anwendung. Um eine Röntgenbild intraoperativ zur besseren Beurteilung zu vergrößern, können verschiedene Prinzipien angewendet werden, Abstandsveränderung zwischen Röhre und Detektor oder Bearbeitung des abgespeicherten Durchleuchtungsbildes („last image hold“) im sog. Postprocessing zur Bildausschnittvergrößerung und Bildbereichverschiebung. Mit der Abstandsänderung des Strahlers von der Körperoberfläche verändert sich das Verhältnis von Austritts- zu Eintrittsdosis. Mit dem Abstand von der Strahlenquelle nimmt die Belastung ab. Das Abstandquadratgesetz verdeutlicht dies: je weiter der Abstand von Operateur und Personal zum Patienten, desto geringer die Streustrahlenbelastung [64]. Das gilt auch für den Patienten selbst.

Ist der Abstand zwischen Patient und Röntgenquelle sehr klein, wird die volle Röntgenleistung auf einen kleineren Bereich der Haut des Patienten gestrahlt. Dies kann das Risiko von Hautverletzungen aufgrund der höheren Hauteingangsdosis erhöhen. Ist der Abstand zwischen Röntgenstrahler und Patient größer, ist nach dem Abstandquadratgesetz eine größere Röntgenleistung erforderlich, um die notwendige Dosis für den Detektor zu erreichen [26]. Aus der Umfrage ergibt sich: 40 % nutzen das Postprocessing, 33 % verwenden die Abstandsveränderung. Damit besteht hier bei einem Drittel der Anwender noch deutliches Potenzial einer weiteren Strahlenreduktion. Die SKT empfiehlt zur intraoperativen Vergrößerung der Röntgenbilder das Postprocessing. Weiterhin sieht die SKT einen Vorteil in der von Anfang an korrekten C‑Bogen-Einstellung und -Positionierung, z. B. mithilfe von Laser-Justierung [16].

### Gepulste Röntgentechnik

Beim *gepulsten* Röntgen wird kein kontinuierlicher Strahl emittiert, sondern ein gepulster, unterbrochener Strahl. Röntgenuntersuchungen mit 15 Bildern/s werden elektronisch zu 30 Bildern erweitert, so entstehen ruckelfreie Betrachtungen [37], auch geringere Frequenzen sind im Gebrauch. Durch Verwendung gepulster Röntgenstrahlen kann die Patientendosis deutlich reduziert werden [23, 37, 39]. Die Reduzierung der Strahlungsabsorptionsdosen durch gepulste Technik wird mit 32 %, mit Low-dose-Einstellung mit 57 % und bei der Kombination gepulst mit Low dose mit 83 % angegeben, verglichen mit dem konventionellen Röntgen am C‑Bogen [14]. Intraoperativ sollte die gepulste statt der kontinuierlichen Fluoroskopie mit möglichst niedriger Wiederholungsrate eingesetzt werden [9]. Die Möglichkeit der gepulsten Röntgentechnik und damit der Strahlenreduzierung wird von den Befragten in 45 % immer, in 23 % häufig und in 17 % nie benutzt. Fast 41 % in der Niederlassung setzen die gepulste Technik nie ein. Hier ist zu fragen, ob evtl. die eingesetzten Geräte diese Technik nicht beherrschen. Die SKT empfiehlt, die gepulste Technik am C‑Bogen standardmäßig einzustellen und Geräte, die diese Technik nicht besitzen, nicht mehr einzusetzen [16].

### Operation auf dem Bildverstärker

Um eine Osteosynthese direkt auf dem *steril abgedeckten Bildverstärker* möglich zu machen, wird die Standardeinstellung des Bildverstärkers – Bildverstärker über dem Op.-Tisch, Röhre unter dem Op.-Tisch – umgekehrt. Hanel et al. empfahlen als Handchirurgen das Operieren direkt auf dem Bildverstärker, um intraoperative Frustrationen mit Gerät zu vermeiden. Man müsse den Bildverstärker nicht mehr intraoperativ bewegen [25].

Insgesamt finden sich nur wenige Studien, die die relative Strahlenexposition bei Verwendung verschiedener räumlicher C‑Bogen-Konfigurationen quantifizieren [17, 27, 62, 67]. Einige Autoren propagieren diese Technik [4, 62]. Andere wollen die Nutzung dieser Anordnung verbieten, da es durch Beschädigung der Aluminiumabdeckung des Bildverstärkers potenziell zu Stromschlag und Explosion kommen kann [68]. Andere sehen Vorteile darin, auf dem Bildverstärker direkt am Ende der Operation projektionsradiographische Röntgendokumentation machen zu können, ohne noch außerhalb des OP diese Röntgendokumentation nach dem Aufwachen zu veranlassen [21]. Heute in vielen Krankenhäusern ist die direkte Einspielung der Bildverstärkerbilder aus dem OP in das PACS der Klinik möglich, und damit entfällt die Röntgenbildgebung außerhalb des OP.

Die Streustrahlenbelastung wird bei inverser Stellung größer gemessen [11, 36]. Die Qualität des BV-Bildes soll hierbei gut sein [62]. Die Strahlungsbelastung der Augen und der Hände des Operateurs steigt und gleichzeitig damit auch der zu durchstrahlende Körperteil im Strahlungsfeld [11, 17]. Die Strahlenbelastung für das Op.-Team ist bei invertierter Stellung des C‑Bogens höher als bei Normalstellung [42, 50]. Besonders bei der suprakondylären kindlichen Humerusfraktur ist die Gefahr groß, dass die Hände des Operateurs im Strahlengang sind, im Durchschnitt zu 13 % [0–97 %] der Röntgenaufnahmen, gemessen in einer Doppelblindstudie [17]. Die Strahlungsdauer in Standardposition des C‑Bogens betrug im Mittel 26 (4–116) s, gegenüber 43 (9–149) s mit invertiertem C‑Bogen [17]. Die Operationszeit wird als gegenüber der Standardposition verlängert berichtet [17]. Das Problem bei den Ergebnissen von Eismann et al. ist, dass sie keinerlei Dosismessungen durchführten, sondern die Anzahl der Röntgenbilder intraoperativ als Grundlage der Auswertung nahmen. Die Ergebnisse von Eismann et al. widersprechen sich mit den Ergebnissen von Tremains. Tremains et al. berichten über eine 59 %ige Strahlenreduktion für die zu operierende auf dem Bildverstärker liegende Hand, auch die Strahlendosis für den Kopf des Operateurs sei um 67 %, des Körperstammes um 45 % und der Schilddrüse um 15 % vermindert [67]. Chapman et al. [11] sahen gegenteilige Ergebnisse mit 3,5fach höherer Strahlenbelastung für die Augen. Hsu et al. sahen 16 % weniger Strahlenbelastung in der Lendenregion, aber 53 % mehr Streustrahlenbelastung in der Halsregion bei invertierter BV-Stellung im Vergleich zum Handtisch; sie schlossen daraus, dass keine Stellung Vorteile habe [27]. Bei invertiertem C‑Bogen ist die Strahlenbelastung in der Leiste des Operateurs erhöht [40]. Aus der Literatur ergibt sich zusammenfassend die Tendenz, dass die Vorteile im Sinne der Strahlenhygiene eher für die konventionelle intraoperative Stellung des C‑Bogens sprechen. In der Umfrage verwenden 63 % diese Technik nie oder 22 % selten. Lediglich 5 % nutzen diese Einstellung des C‑Bogens regelhaft. Die SKT steht dieser Operationstechnik auf der Bildverstärkeroberfläche aus Gründen der Strahlenhygiene ablehnend gegenüber [16].

### Röntgenkontrollen

#### Nach Metallentfernung

Nach unfallchirurgischen Operationen war früher auch bei Kindern Standard, bei fast jeder Kontrollvorstellung zu röntgen [49], auch bei Kindern. Garg et al. empfehlen, dass im Rahmen der Metallentfernung nach suprakondylärer Humerusfraktur bei unauffälligen Kindern auf Röntgenaufnahmen vor der Pin-Entfernung verzichtet wird. So können Strahlung und auch Kosten vermieden werden, denn bei 1213 Patienten ergaben sich aus den präoperativen Röntgenaufnahmen vor der Metallentfernung keine interventionsbedürftigen Erkenntnisse [20]. Der SKT-Konsens bezieht klar Position: Vor der Metallentfernung soll die knöcherne Konsolidierung zeitnah vor der Metallentfernung radiologisch gesichert werden [16]. Intraoperativ soll regelhaft auf das Röntgen verzichtet werden, die Vollständigkeit der Metallentfernung im Operationsbericht dokumentiert werden [16]. Postoperativ soll nach unproblematischem Verlauf keinerlei Röntgenuntersuchung erfolgen [16]. Das bei z. B. überwachsenen Implantaten intraoperativ das Röntgen sinnvoll sein kein, wird nicht infrage gestellt.

Das Spektrum der Aussagen zu Röntgendokumentation und radiologischen Kontrollen ist in der Umfrage vielfältig. 24,5 % verzeichnen auf jegliche postoperative Röntgenkontrollen, 23 % sagen, dass die intraoperative Bildgebung immer ausreichend, 31,5 % häufig ausreichend sei. Die Kinderchirurgen verzichten in 76 % auf postoperative Röntgenkontrollen oder -dokumentationen. Insgesamt sollten aufgrund fehlender Konsequenzen die Röntgenaufnahmen nach Metallentfernung nur in begründbaren seltenen Fällen erwogen werden.

### Verlaufskontrollen

Nach *Bohrdrahtosteosynthese* einer suprakondylären Humerusfraktur zeigt sich kein einheitliches Vorgehen in der Umfrage. Während 32 % direkt postoperativ röntgen, fordern 30 % noch einmal eine Röntgenkontrolle direkt vor der Entlassung. Danach wird zu unterschiedlichen Häufigkeiten nach einer, 2, 4, 6 Wochen und vor der Metallentfernung geröntgt. 33 % röntgen postoperativ und direkt vor der Metallentfernung. Vorausgesetzt wurde eine stabile, intraoperativ kontrollierte Osteosynthese. Karalius kommt in seiner Arbeit zu dem Schluss, dass z. B. Röntgenaufnahmen bei Gartland-Typ-III-Frakturen innerhalb von 7 bis 10 Tagen postoperativ durchgeführt werden und nur dann wiederholt werden sollten, wenn Indikatoren auftreten, wie z. B. anhaltenden Schmerzen [31]. Klinischer Nutzen und Konsequenzen aus postoperativen Röntgenbildern ohne Vorliegen klinischer Auffälligkeiten lassen sich nicht finden [73]. Andere Autoren sehen von einer Röntgenverlaufskontrolle bis zu Pin-Entfernung regelhaft ab, da leichte Achsabweichungen oder Pin-Migration so gut wie nie zu Konsequenzen führten [32, 74]. Mansur et al. sehen bei einer adäquaten intraoperativen Fixierung erst nach 3 Wochen zur Metallentfernung eine Röntgendokumentation als notwendig an [38]. Andere sehen auch diese Kontrolluntersuchung als anfechtbar an [61]. Die SKT empfiehlt intraoperativ zur Dokumentation 2 Standardebenen [16]. Nur bei fehlender Dokumentationsmöglichkeit der intraoperativen Bilder im PACS erfolgt eine Kontrolle innerhalb einer Woche postoperativ, gefolgt von einer Röntgenstandarduntersuchung vor Metallentfernung nach 4 bis 6 Wochen [16].

### Grünholzfrakturen

Einige Autoren sind der Meinung, dass Grünholzfrakturen des Unterarms sehr selten erneute Intervention nach geschlossener Reposition benötigen [66]. Die inkompletten Frakturen vom Typ Grünholz, die konservativ behandelt werden, haben Tendenz instabil zu werden und innerhalb der ersten 2 Wochen zu dislozieren [53]. Deshalb werden Röntgenkontrollen für diesen Zeitraum oder auch sonographische Kontrollen empfohlen. Mit Reduzierung der Kontrollfrequenz wird die Strahlenexposition bei 2 Kontrollaufnahmen im Gipsverband um 41 % vermindert [66]. Die Ultraschallkontrolluntersuchung ist möglich, die Strahlenbelastung auf null zu reduzieren und dennoch mit hoher Sensitivität und Spezifität die Frakturen zu beurteilen [10, 12, 70].

Es findet sich bei den Befragten hinsichtlich der Grünholzfraktur ein gewisser Rhythmus der posttraumatischen Kontrollen mit 2 Ebenen nach einer Woche mit 37 %, nach 2 Wochen mit 11 % und 4 Wochen mit 28 %. 40 % aller Befragten röntgen nach dieser Fraktur gar nicht. Hier führen die Niedergelassen mit 49 % bei dem Verzicht auf Röntgenkontrollen. Die Grünholzfraktur ist im Vergleich zur Stauchungsfraktur immer als semistabil einzustufen und bedarf der Kontrolle mithilfe der Sonographie oder des Röntgens. Dem SKT-Konsens ist zu entnehmen, das bei distalen, metaphysären Radius-Grünholz-Frakturen bei unter 10-jährigen Kindern nach einer Woche in 2 Ebenen im Röntgen die Stellung kontrolliert werden soll [16]. Nach Cast-Abnahme nach 4 Wochen erfolgt die Dokumentation der Konsolidierung in 2 Ebenen in der Projektionsradiographie [16]. Diese Röntgenkontrollen sollen nur erfolgen, wenn grundsätzlich die mögliche Notwendigkeit einer aktiven Stellungsverbesserung angenommen wird [16].

### Claviculafrakturen

Claviculafrakturen machen rund 70 % aller Frakturen bei Kindern [58].

Claviculaschaftfrakturen heilen überwiegend konservativ problemlos aus [24, 43, 48, 48, 59]. Calder et al. raten nach ihren Studien von Röntgenkontrollen bei der unkomplizierten Claviculafraktur ab [8]. Die röntgenologische Beurteilung der knöchernen Heilung dieser Frakturen wird als schwierig und potenziell unzuverlässig eingestuft [59]. O’Neill sieht Röntgenkontrollen gar als unnütz an, wenn klinische Auffälligkeiten fehlen [43], Adamich sieht sie bei konservativer Behandlung als nicht notwendig an [2]. Die klinische Verlaufskontrolle sollte ausreichend sein [35]. In Studien wird die Diagnostik mit Ultraschall außerdem höhenwertig eingestuft als die Röntgenuntersuchung [1]. Unnötige Strahlenbelastung bei Claviculafrakturen, insbesondere bei Kindern, sollten vermieden werden [13, 34].

Bei einer unverschobenen Schaftfraktur des Schlüsselbeins sehen fast 55 % aller Befragten keine Indikation zu Röntgenkontrollen, die Kinderchirurgen in 87 %. Einige der Befragten sehen weitere Röntgenkontrollen als notwendig an, nach einer Woche in einer Ebene 10,4 %, nach 2 Wochen in 2 Ebenen 14 %, nach 4 Wochen in 2 Ebenen 12,5 %. Die SKT sieht insgesamt die Indikation und Notwendigkeit von ausführlicher Röntgenuntersuchung bei Claviculafrakturen sehr zurückhaltend [16]. Sie sieht eine weite Ebene in der primären Röntgendiagnostik als nicht zwingend an [16]. Eine Verlaufskontrolle mit Röntgen lehnt sie ab, denn ohne therapeutische Konsequenz bestehe keine Indikation zur radiologischen Verlaufsdokumentation [16].

### Tibiaschaftfrakturen

Bei einer unverschobenen konservativ behandelten Tibiaschaftfraktur empfehlen 63 % eine Kontrolle nach einer Woche in 2 Ebenen, 24 % nach 2 Wochen, 37 % nach 4 Wochen und 32 % nach 6 Wochen. Auch bei dieser Fraktur erscheint die klinische Verlaufskontrolle ausreichend [35]. Nach Gipsabnahme kann normalerweise entstandener Kallus vorsichtig palpiert werden [35]. Andere Autoren empfehlen nach 2 Wochen eine Röntgenkontrolle im Gips, um Frakturdislokationen auszuschließen [47, 52]. Bei einer nichtdislozierten Fraktur, die also nicht reponiert werden musste, ist nach allgemeiner Erfahrung das Dislokationsrisiko sehr gering.

Die Sektion Kindertraumatologie sieht in ihrem Konsens für undislozierte, isolierte Tibiaschaftspiralfrakturen und konservativer Behandlung eines 6–10jährigen Kindes Röntgenkontrollen nur nach einer Woche und nach 4 Wochen vor [16]. Der Zeitpunkt eine Woche gilt für die rechtfertigende Fragestellung nach dem möglichen Auftreten einer zu behebenden, sekundären Varisierung, nach 4 Woche der Konsolidierung und Einschätzung der Belastbarkeit [16]. Da Kinder, wenn sie schmerzfrei sind, von sich aus meistens schon belasten, kann individuell auf die 4‑Wochen-Aufnahme auch verzichtet werden.

## Fazit

Die Erhebung des aktuellen Stands beim Einsatz bildgebender Verfahren im OP und in der Verlaufskontrolle im Kindes- und Jugendalter ergibt, dass keine einheitliche standardisierte Vorgehensweise bei der Indikation und Anwendung der Bildgebung besteht.

Die Möglichkeiten der Strahlenreduktion bei der intraoperativen Anwendung werden lt. der Umfrage nicht komplett ausgenutzt, so die Kollimation, das Postprocessing, das gepulste Röntgen. Kontrollen nach konservativer Behandlung und operativen Verfahren werden teilweise in hoher Frequenz eingesetzt. Ob klinische Konsequenzen aus dieser Bildgebung gezogen wurden oder werden konnten, war nicht Thema der Umfrage, erscheint aber z. B. bei der Claviculafraktur eher unwahrscheinlich.

Es bleibt weiterhin die Aufgabe für alle, die in der Kindertraumatologie tätig sind, zu sensibilisieren, um den Strahlenschutz für unsere jungen Patienten zu optimieren. In der Zukunft sollten Algorithmen erarbeitet werden, die auf evidenzbasierten Grundlage, Vorschläge zur effektiven Bildgebung in der Kindertraumatologie sowohl bei konservativer als auch operativer Therapie geben. Solche Handlungsanweisungen können zur Reduktion des Einsatzes der ionisierenden Strahlen, aber auch zur Absicherung der behandelnden Ärzte beitragen.

## Footnotes

1 Aus Gründen der besseren Lesbarkeit und Verständlichkeit des Textes wird hier das generische Maskulinum als geschlechtsneutrale Form verwendet. Diese Form impliziert immer alle Geschlechter.
